# Clinical Features and Preventability of Delayed Diagnosis of Pediatric Appendicitis

**DOI:** 10.1001/jamanetworkopen.2021.22248

**Published:** 2021-08-31

**Authors:** Kenneth A. Michelson, Scott D. Reeves, Joseph A. Grubenhoff, Andrea T. Cruz, Pradip P. Chaudhari, Arianna H. Dart, Jonathan A. Finkelstein, Richard G. Bachur

**Affiliations:** 1 Division of Emergency Medicine, Boston Children’s Hospital, Boston, Massachusetts; 2 Division of Pediatric Emergency Medicine, Department of Pediatrics, Cincinnati Children's Hospital Medical Center, Cincinnati, Ohio; 3 Section of Pediatric Emergency Medicine, University of Colorado School of Medicine, Aurora; 4 Children's Hospital Colorado, Aurora; 5 Department of Pediatrics, Baylor College of Medicine, Houston, Texas; 6 Division of Emergency and Transport Medicine, Children's Hospital Los Angeles, Los Angeles, California; 7 Keck School of Medicine of the University of Southern California, Los Angeles; 8 Division of General Pediatrics, Boston Children’s Hospital, Boston, Massachusetts

## Abstract

**Importance:**

Delayed diagnosis of appendicitis is associated with worse outcomes than timely diagnosis, but clinical features associated with diagnostic delay are uncertain, and the extent to which delays are preventable is unclear.

**Objective:**

To determine clinical features associated with delayed diagnosis of pediatric appendicitis, assess the frequency of preventable delay, and compare delay outcomes.

**Design, Setting, and Participants:**

This case-control study included 748 children treated at 5 pediatric emergency departments in the US between January 1, 2010, and December 31, 2019. Participants were younger than 21 years and had a diagnosis of appendicitis.

**Exposures:**

Individual features of appendicitis and pretest likelihood of appendicitis were measured by the Pediatric Appendicitis Risk Calculator (pARC).

**Main Outcomes and Measures:**

Case patients had a delayed diagnosis of appendicitis, defined as 2 emergency department visits leading to diagnosis and a case review showing the patient likely had appendicitis at the first visit. Control patients had a single emergency department visit yielding a diagnosis. Clinical features and pARC scores were compared by case-control status. Preventability of delay was assessed as unlikely, possible, or likely. The proportion of children with indicated imaging based on an evidence-based cost-effectiveness threshold was determined. Outcomes of delayed diagnosis were compared by case-control status, including hospital length of stay, perforation, and multiple surgical procedures.

**Results:**

A total of 748 children (mean [SD] age, 10.2 [4.3] years; 392 boys [52.4%]; 427 White children [57.1%]) were included in the study; 471 (63.0%) had a delayed diagnosis of appendicitis, and 277 (37.0%) had no delay in diagnosis. Children with a delayed diagnosis were less likely to have pain with walking (adjusted odds ratio [aOR], 0.16; 95% CI, 0.10-0.25), maximal pain in the right lower quadrant (aOR, 0.12; 95% CI, 0.07-0.19), and abdominal guarding (aOR, 0.33; 95% CI, 0.21-0.51), and were more likely to have a complex chronic condition (aOR, 2.34; 95% CI, 1.05-5.23). The pretest likelihood of appendicitis was 39% to 52% lower in children with a delayed vs timely diagnosis. Among children with a delayed diagnosis, 109 cases (23.1%) were likely to be preventable, and 247 (52.4%) were possibly preventable. Indicated imaging was performed in 104 (22.0%) to 289 (61.3%) children with delayed diagnosis, depending on the imputation method for missing data on white blood cell count. Patients with delayed diagnosis had longer hospital length of stay (mean difference between the groups, 2.8 days; 95% CI, 2.3-3.4 days) and higher perforation rates (OR, 7.8; 95% CI, 5.5-11.3) and were more likely to undergo 2 or more surgical procedures (OR, 8.0; 95% CI, 2.0-70.4).

**Conclusions and Relevance:**

In this case-control study, delayed appendicitis was associated with initially milder symptoms but worse outcomes. These findings suggest that a majority of delayed diagnoses were at least possibly preventable and that many of these patients did not undergo indicated imaging, suggesting an opportunity to prevent delayed diagnosis of appendicitis in some children.

## Key Points

**Question:**

Which clinical features are associated with delayed diagnosis of pediatric appendicitis, and to what extent are delays preventable?

**Findings:**

In this case-control study of 748 children, those who experienced delayed diagnosis had less clinically apparent symptoms; among children for whom imaging would have been cost-effective, 22% to 61% received it.

**Meaning:**

These findings suggest that undertesting for appendicitis may account for a considerable fraction of cases of delayed diagnosis of appendicitis.

## Teaser

This case-control study assesses the clinical features associated with a delayed diagnosis of pediatric appendicitis and whether a delayed diagnosis can be prevented.

## Introduction

Appendicitis is the most common surgical emergency in children.^1^ Significant delays in diagnosis are associated with worse outcomes, particularly perforated appendicitis.^2,3,4^ Perforated appendicitis is associated with substantial morbidity, including abdominal abscess, small bowel obstruction, sepsis, bowel resection, longer hospital stays, increased encounters with health care, and higher cost.^5,6,7,8^ Delayed diagnosis of appendicitis is also a top cause of malpractice claims in pediatrics.^9^

Delayed diagnosis of appendicitis in the emergency department (ED) is frequently associated with an incorrect diagnosis during an earlier visit to care.^10,11,12^ Misdiagnosed conditions include constipation, gastroenteritis, or symptom-based diagnoses, such as vomiting or diarrhea.^11,13,14^ Imaging may decrease the likelihood of a misdiagnosis, but overuse of computed tomography (CT) increases radiation-related risks, and use of ultrasonography in patients with a low probability of appendicitis has a high rate of both false positives and inconclusive results.^15,16,17^ Although duration of symptoms, age, and sex are known to be associated with the likelihood of a delayed diagnosis, the specific symptom and care patterns associated with delays are unclear.^11,13,18^

Understanding which patients are at risk of delayed diagnosis would aid efforts to prevent such delays. Therefore, we performed a case-control study comparing children with delayed diagnosis with children with timely diagnosis of appendicitis to assess clinical features that are associated with delay and elements of care that are associated with potentially preventable delays.

## Methods

### Design and Data Sources

We conducted a case-control study of children and young adults younger than 21 years with appendicitis. Potential cases involved a delayed diagnosis of appendicitis, defined as 2 ED visits within 7 days leading to diagnosis and a case review showing the patient likely had appendicitis at the first visit. Cases were drawn from a cohort of patients treated for appendicitis who visited 1 of 5 US pediatric EDs from January 1, 2010, to December 31, 2019, identified in the Pediatric Health Information System database. The Pediatric Health Information System contains information about patient demographic details, medications, diagnoses, radiology, and procedures for freestanding pediatric hospitals in the US. These records are linkable to local electronic medical records, which allows for detailed case review.

Control patients were children with a timely diagnosis of appendicitis at their first ED visit and were identified from a prospective study of children with appendicitis that included children from a single pediatric ED between February 26, 2010, and April 20, 2013.^17^ Controls were identified at the time of presentation, and treating clinicians completed a demographic and symptom survey. The control ED was 1 of the 5 sites used to identify cases. Unlike most case-control studies, we anticipated that we would enroll fewer controls than cases because of the sample size needs of the original case and control studies.

Appendicitis was defined as either undergoing appendectomy or, if managed nonoperatively, as imaging evidence of appendicitis followed by improvement with antibiotic therapy and/or procedures such as abdominal abscess drainage. Case patients were excluded if there were insufficient records, if the patient left without being seen on the first (ie, index) encounter, if the patient was transferred on the index encounter, or if the case was judged not to have been delayed. Control patients were excluded if there were insufficient records to perform a case review. Institutional review boards at each site approved this study with a waiver of informed consent because of minimal risk to participants. We adhered to the Strengthening the Reporting of Observational Studies in Epidemiology (STROBE) guidelines for reporting case-control studies.^19^

### Outcomes

The main outcome was delayed diagnosis of appendicitis. Control patients (those without a delayed diagnosis) had a single ED encounter leading to an appendicitis diagnosis. All participants with a potentially delayed diagnosis underwent review of documentation from both the index and the second encounter (when the diagnosis was made, ie, the delay encounter). Delayed diagnosis was defined by reviewer determination that appendicitis was probably or near-definitely present but not diagnosed during the index encounter (definitions in eTable 1 in the Supplement). This definition was developed through a multicenter, multispecialty expert consensus process.^20^ Case review and data abstraction were performed by board-certified pediatric emergency medicine attending physicians (J.A.G., S.D.R., P.P.C., A.T.C., and K.A.M.). One reviewer from each site was trained through supervised review of 80 case vignettes. Reviewers (J.A.G., S.D.R., P.P.C., A.T.C., and K.A.M.) were coauthors of this study but were unaware of the study hypothesis. We did not perform interrater reliability assessments, as our pilot study showed high interrater reliability in the assessment of delayed diagnosis.^21^

A secondary outcome among delayed diagnosis cases was the change in clinical discernibility of appendicitis between the index and delayed encounters. Discernibility was measured using 2 validated clinical scoring systems: the Pediatric Appendicitis Score (PAS) and the Pediatric Appendicitis Risk Calculator (pARC).^22,23^ The PAS score is measured on a scale of 0 to 10, with higher scores indicating a greater likelihood of appendicitis. The pARC score estimates the likelihood of appendicitis on a scale of 0% to 100%. Both the PAS and pARC scores rely on peripheral white blood cell (WBC) and neutrophil counts. However, the WBC count was not obtained in many cases during the index encounter. Therefore, in evaluations involving the PAS and pARC, we determined the scores using several approaches: (1) analyzed only cases with laboratory values present; (2) imputed missing WBC values from all other clinical variables; and (3) imputed missing WBC values as 5000, 10 000, 15 000, or 20 000 cells per microliter. A complete description appears in the eMethods in the Supplement.

Cases were also reviewed for the secondary outcome of preventable delayed diagnosis, defined as a missed opportunity to improve diagnosis (MOID).^24^ To grade the likelihood of a MOID, we used the validated Modified Safer Dx checklist.^25^ The Safer Dx measures the likelihood of a MOID using 13 items relating to individual elements of an encounter that could lead to MOIDs. The first 12 items include the history, physical examination, data gathering, presence of “red flags,” incomplete caregiver information, completeness of evaluation, diagnostic reasoning, test interpretation, follow-up of results, differential formation, disease evolution, and how closely the index presentation reflected typical features of the disease. The final item, the MOID score, graded the overall likelihood of a MOID. All items are measured on a scale of 1 to 7. We further classified a score of 1 to 2 as an unlikely MOID, 3 to 5 as a possible MOID, and 6 to 7 as a likely MOID.

### Variables

Potential clinical features associated with delayed diagnosis were chosen a priori based on clinical features related to the ultimate diagnosis of appendicitis from previous literature.^22,23^ These features included age, sex, duration of abdominal pain, anorexia, nausea or vomiting, migration of pain to the right lower quadrant, history of fever, pain with walking, maximal pain in the right lower quadrant, and abdominal guarding. If the symptom was not mentioned in the record, it was assessed as being absent, as is typical for this type of study.^26^ We also assessed the presence of a complex chronic condition based on same- and previous-encounter diagnosis codes.^27^

We hypothesized that several care features would be associated (positively or negatively) with delayed diagnosis, including obtaining ultrasonographic imaging, obtaining a CT scan, use of a nonopioid analgesic (acetaminophen, ibuprofen, ketorolac, or naproxen), use of an opioid analgesic (fentanyl, hydromorphone, morphine, or oxycodone), or use of ondansetron. Finally, because we planned to evaluate disparities between timely and delayed diagnosis, we collected race, ethnicity, and insurance information as recorded in the source database.

### Statistical Anlysis

#### Primary Analysis

For the primary analysis, we summarized the proportion of delayed cases that were MOIDs. Among cases, we evaluated which elements of care were associated with preventable delay by performing Spearman correlations between each Safer Dx item and the overall MOID score. Correlations were considered strong with a ρ greater than 0.7 and moderate at a ρ of 0.5 to 0.7. Previous research has suggested that imaging is cost-effective if the probability of appendicitis is at least 16%.^28^ We evaluated the proportion of children who underwent indicated imaging testing (ultrasonography or CT) with an index encounter pARC appendicitis probability of at least 16%.

#### Secondary Analysis

For the secondary analysis, we first compared the characteristics of case and control patients using χ^2^ tests. To assess the likelihood of selection bias affecting analysis of differences between cases and controls, we compared the characteristics of cases from the single control site to cases from other study sites. An absence of observable differences would suggest a low risk of selection bias.

Next, we developed 2 logistic regression models to examine features associated with delayed diagnosis. In model 1, we included all clinical features. In model 2, we evaluated how features of care are associated with delayed diagnosis by including all care features after adjusting for all clinical features.

We hypothesized that cases of delayed diagnosis would be less clinically discernible during the index encounter than controls, so we plotted and compared PAS and pARC scores between cases and controls using Wilcoxon rank sum tests. We then evaluated how clinical features of appendicitis progress in delayed diagnosis cases. Hypothesizing that cases with a delay would become more discernible as time passed, we determined the difference in median PAS and pARC scores between index and delay encounters and applied a Wilcoxon signed rank test. Finally, we quantified the likelihood that a clinical feature would develop between the 2 ED encounters using conditional logistic regression using encounter (delay vs index) as the outcome and clinical features as covariates, and conditioned on the patient. This analysis resulted in estimates of the odds ratio (OR) for a feature being present at the delay encounter compared with the index encounter in a particular patient.

We planned to evaluate disparities in delayed diagnosis by race, ethnicity, and insurance type; however, we found that the distribution of these characteristics varied too greatly between the source populations of case and control patients. Therefore, we were unable to perform this analysis.

Length of hospitalization, presence of perforated appendicitis, and whether patients underwent appendectomy were determined for cases by likelihood of MOID and for controls. Appendectomy status was determined by having a procedure code for the procedure (*International Classification of Diseases, Ninth Revision* [*ICD-9*] 47.01 or 47.09; *International Statistical Classification of Diseases and Related Health Problems, Tenth Revision* [*ICD-10*] 0DTJ4ZZ or 0DTJ0ZZ). We also determined the proportion of patients with multiple abdominal surgical procedures, defined as an abdominal surgical procedure on more than 1 hospital day. These procedures included appendectomy as defined above and abdominal abscess drainage (*ICD-9* 47.2 or 54.91; *ICD-10* stems 0D9J, 0D9W, or 0W9F-0W9J). Outcomes were reported using the mean and 95% CI or proportion and 95% binomial exact CI. Differences between groups were reported using a 95% CI of the difference in means or the conditional maximum likelihood estimate OR. Secondary analyses did not adjust for multiple comparisons and thus were considered exploratory. Statistical significance was defined as *P* < .05, and all *P* values were 2-sided. Analyses were performed in R, version 4.0.2 (R Foundation).

## Results

There were 801 children with delayed diagnosis and 294 children with timely diagnosis of appendicitis. Among those with a delayed diagnosis, 19 were excluded for insufficient records, 32 for leaving without being seen from the index encounter, 81 for transfer from the index encounter, and 198 for not having a delayed diagnosis upon case review. In addition, 17 children with a timely diagnosis were excluded for insufficient records. After exclusions, a total of 748 children (mean [SD] age, 10.2 [4.3] years; 392 boys [52.4%] and 356 girls [47.6%]; 427 White children [57.1%]) were included in the study; 471 (63.0%) had a delayed diagnosis of appendicitis, and 277 (37.0%) had no delay in diagnosis. Of the total cohort, 55 children (7.4%) had a complex chronic condition (Table 1).

**Table 1.** Demographic Features of Cases and Controls

Variable	No. (%)	
	Delayed diagnosis (n = 471)	Timely diagnosis (n = 277)
Age, y		
<5	82 (17.4)	16 (5.8)
5-9	185 (39.3)	83 (30.0)
10-14	145 (30.8)	114 (41.2)
15-21	59 (12.5)	64 (23.1)
Male	231 (49.0)	161 (58.1)
Female	240 (51.0)	116 (41.9)
Race		
White	234 (49.7)	193 (73.9)
Black	34 (7.2)	10 (3.8)
Asian	3 (0.6)	8 (3.1)
American Indian or Alaska Native	0	1 (0.4)
Native Hawaiian or Pacific Islander	1 (0.2)	0
Other^a^	199 (42.3)	49 (18.8)
Ethnicity		
Hispanic or Latino	288 (61.1)	36 (13.1)
Not Hispanic or Latino	175 (37.2)	238 (86.9)
Unknown	8 (1.7)	0
Insurance		
Public	296 (62.8)	43 (15.6)
Private	156 (33.1)	230 (83.3)
Self-pay	15 (3.2)	0
Unknown	4 (0.8)	3 (1.1)
Complex chronic condition	41 (8.7)	14 (5.1)
Imaging completed on initial encounter		
Computed tomography scan	15 (3.2)	81 (29.2)
Magnetic resonance imaging	3 (0.6)	1 (0.4)
Ultrasonography	97 (20.6)	228 (82.3)
Interval between index and delay encounters, median (IQR)	1 (1-2)	NA

Abbreviations: IQR, interquartile range; NA, not applicable.

^a^ Other race included multiracial patients and those for whom hospitals listed the race as “other.”

There were few differences between the delayed and timely diagnosis groups in terms of age, sex, complex chronic condition, revisit interval, or index encounter symptoms except for anorexia and migratory pain (eTable 2 in the Supplement). Race, ethnicity, and insurance type each differed significantly. Thus, we regarded selection bias to be limited between cases and controls except with regard to sociodemographic factors. The 5 most common diagnoses given at the index encounter of delayed diagnosis cases were gastroenteritis (132 of 471 [28.0%]), abdominal pain (81 of 471 [17.2%]), constipation (79 of 471 [16.8%]), urinary tract infection (27 of 471 [5.7%]), and viral syndrome (24 of 471 [5.1%]).

### Primary Analysis

Among cases of delayed diagnosis of appendicitis, 109 (23.1%) were unlikely MOIDs, 247 (52.4%) were possible MOIDs, and 115 (24.4%) were likely MOIDs; in other words, there were possible or probable opportunities to improve diagnosis for 76.8% of patients with a delayed diagnosis. Three of 12 Safer Dx elements were correlated with the overall likelihood of a MOID (eTable 3 in the Supplement): history should have prompted additional diagnostic evaluation (Spearman ρ = 0.77), diagnostic reasoning was not appropriate (ρ = 0.77), and clinical presentation was mostly typical of the ultimate diagnosis (ρ = 0.76). One Safer Dx element was correlated with MOID: alarm symptoms or red flags were not acted upon (ρ = 0.65). The number of patients who received indicated imaging was between 104 of 471 (22.0%; 95% CI, 17.9%-26.5%) and 289 of 471 (61.3%; 95% CI, 49.4%-72.4%) depending on the WBC imputation method.

### Secondary Analysis

In a model assessing clinical features at the index encounter, patients with delayed diagnosis of appendicitis were more likely to have had abdominal pain for 48 to 96 hours (adjusted OR [aOR], 2.40; 95% CI, 1.16-4.97) compared with less than 24 hours, no pain with walking (aOR, 0.16; 95% CI, 0.10-0.25), abdominal pain maximally outside the right lower quadrant (aOR, 0.95; 95% CI, 0.57-1.58), no abdominal guarding (aOR, 0.33; 95% CI, 0.21-0.51), and a complex chronic condition (aOR, 2.34; 95% CI, 1.05-5.23) (Table 2). Adding care features to the model, children who received nonsteroidal anti-inflammatory drugs (aOR, 3.78; 95% CI, 2.10-6.80) or ondansetron (aOR, 1.99; 95% CI, 1.10-3.61) were more likely to have a delay in diagnosis, whereas those who received opioids (aOR, 0.26; 95% CI, 0.14-0.49), a CT scan (aOR, 0.10; 95% CI, 0.05-0.22), or ultrasonography (aOR, 0.13; 95% CI, 0.08-0.24) were less likely. In this model, the presence of a complex chronic condition was no longer associated with delay.

**Table 2.** Models Revealing Clinical Features Associated With Delayed Diagnosis of Appendicitis Compared With Those Without Delay

Covariate	aOR (95% CI)	
	Model 1: clinical factors^a^	Model 2: clinical and care factors
**Clinical features from the index encounter**		
Age, y		
<5	1 [Reference]	1 [Reference]
5-9	0.54 (0.24-1.20)	0.63 (0.24-1.65)
10-14	0.48 (0.20-1.15)	0.68 (0.23-2.00)
>14	0.72 (0.33-1.58)	0.70 (0.27-1.84)
Male sex	0.93 (0.61-1.43)	0.82 (0.49-1.38)
Female sex	1 [Reference]	1 [Reference]
Complex chronic condition	2.34 (1.05-5.23)	2.23 (0.89-5.60)
Abdominal pain duration, h		
<24 Or unknown	1 [Reference]	1 [Reference]
24-47	1.28 (0.77-2.14)	1.56 (0.81-2.99)
48-96	2.40 (1.16-4.97)	2.65 (1.11-6.35)
>96	2.15 (0.97-4.77)	1.40 (0.57-3.47)
Anorexia	0.82 (0.52-1.30)	1.26 (0.72-2.22)
Fever	0.78 (0.48-1.26)	0.55 (0.30-1.02)
Nausea or vomiting	1.57 (0.96-2.55)	1.16 (0.64-2.11)
Pain with walking	0.16 (0.10-0.25)	0.16 (0.09-0.28)
Maximal pain in the right lower quadrant	0.12 (0.07-0.19)	0.27 (0.15-0.50)
Migration of pain to the right lower quadrant	0.95 (0.57-1.58)	1.03 (0.57-1.85)
Abdominal guarding	0.33 (0.21-0.51)	0.50 (0.29-0.88)
**Care features from the index encounter**		
NSAID	NA	3.78 (2.10-6.80)
Opioid		0.26 (0.14-0.49)
Ondansetron		1.99 (1.10-3.61)
CT scan		0.10 (0.05-0.22)
Ultrasonography		0.13 (0.08-0.24)

Abbreviations: aOR, adjusted odds ratio; CT, computed tomography; NA, not applicable; NSAID, nonsteroidal anti-inflammatory drug.

^a^ In model 1, we included as covariates clinical factors. In model 2, clinical factors were supplemented with care factors.

**Figure 1.**
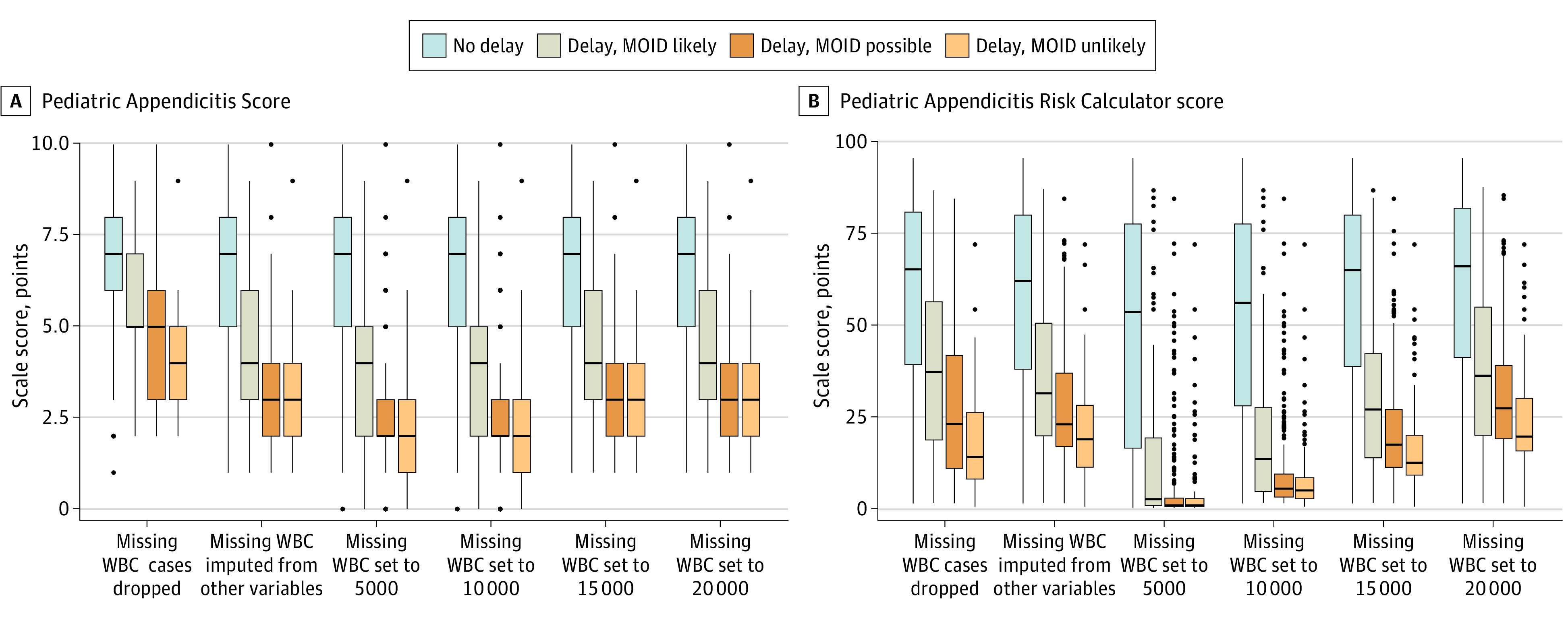
Pediatric Appendicitis Score and Pediatric Appendicitis Risk Calculator Score Among Children With and Without a Delay in Diagnosis From the Index (First) Encounter Variability in scores is depicted using standard box plots with the boxes depicting the median, 25th, and 75th percentile scale scores, the standard whisker lengths, and dots representing outliers. Those with a delay were further stratified by the likelihood of a missed opportunity to improve diagnosis. Scores are presented depending on the handling of missing peripheral white blood cell (WBC) counts. MOID indicates missed opportunity to improve diagnosis.

The index encounter PAS and pARC scores were lower in children who experienced a delayed diagnosis of appendicitis compared with those with a timely diagnosis (Figure 1). White blood cell values were missing from 53 of 277 index encounters (19.1%) among children with timely diagnosis and 358 of 471 index encounters (76.0%) among children with delayed diagnosis. Regardless of WBC imputation method, PAS and pARC scores were significantly higher from the index encounters of children with timely rather than delayed diagnosis of appendicitis: the median PAS scores differed by 2 to 4 points depending on imputation method, and the median pARC scores differed by 39 to 52 percentage points (*P* < .001 for each comparison of cases to controls).

**Figure 2.**
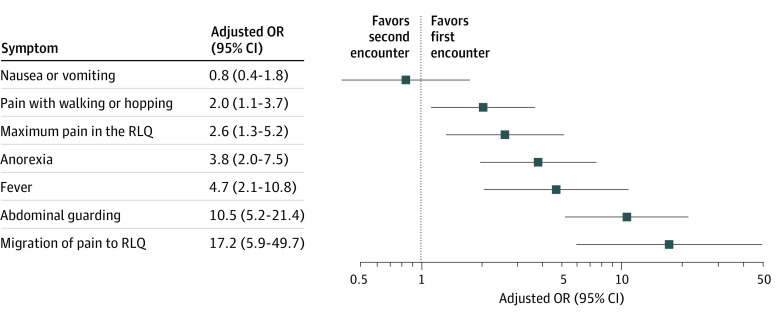
Forest Plot of Sign or Symptom Being Present at Second vs First Encounter Progression of symptoms and signs of appendicitis is shown as the relative likelihood of being present at the delay vs index encounter among children experiencing a delayed diagnosis. OR indicates odds ratio; RLQ, right lower quadrant.

Among children with a delayed diagnosis of appendicitis, the increase in median PAS score between index and delay encounters ranged from 1 to 4 points depending on WBC imputation method (*P* < .001 for each comparison). The increase in median pARC scores ranged from 12 to 43 percentage points (*P* < .001 for each comparison). Each clinical feature was more likely to be present at the delay encounter compared with the index encounter except for nausea or vomiting (Figure 2). The symptom most likely to have developed between encounters was migration of pain to the right lower quadrant (aOR, 17.2; 95% CI, 5.9-49.7).

Outcomes of appendicitis were generally worse among children with delayed vs timely diagnosis (Table 3). Children with delayed diagnosis had longer hospital length of stay (mean difference, 2.8 days; 95% CI, 2.3-3.4 days), higher perforation rates (OR, 7.8; 95% CI, 5.5-11.3), a decreased likelihood of appendectomy at the time of ultimate diagnosis (OR, 0.33; 95% CI, 0.19-0.55), and a higher likelihood of undergoing 2 or more abdominal surgical procedures (OR, 8.0; 95% CI, 2.0-70.4) compared with children with timely diagnosis.

**Table 3.** Outcomes of Delayed Diagnosis of Appendicitis

Outcome	Value (95% CI)				
	Controls (n = 277)	Cases			
		All cases (n = 471)	Unlikely MOID (n = 109)	Possible MOID (n = 247)	Likely MOID (n = 115)
Mean hospitalization time, d	2.2 (1.9-2.5)	5.0 (4.5-5.5)	4.0 (3.3-4.7)	5.5 (4.7-6.4)	5.0 (4.3-5.6)
Perforation, %	22.7 (17.9-28.1)	69.9 (65.5-74.0)	53.2 (43.4-62.8)	74.9 (69.0-80.2)	74.8 (65.8-82.4)
Appendectomy, %	92.8 (89.1-95.5)	80.7 (76.8-84.1)	78.9 (70.0-86.1)	81.0 (75.5-85.7)	81.7 (73.5-88.3)
≥2 Surgical procedures, %	0.7 (0.2-1.8)	5.5 (3.6-8.0)	2.8 (0.6-7.8)	8.1 (5.0-12.2)	2.6 (0.5-7.4)

Abbreviation: MOID, missed opportunity to improve diagnosis.

Outcomes were taken from the encounter in which appendicitis was diagnosed and managed (the index encounter from controls and the delay encounter from cases).

## Discussion

In this case-control study, children with delayed diagnosis at their index ED encounter were more likely to have a longer duration of abdominal pain, no pain with walking, abdominal pain outside the right lower quadrant, no abdominal guarding, and a complex chronic condition compared with children with a timely diagnosis of appendicitis. Based on PAS or pARC scores, children with delayed diagnoses were less clinically discernible. They were more likely to receive nonsteroidal anti-inflammatory drugs and antiemetic therapy and less likely to receive opioids or undergo imaging. In 76.8% of patients with a delayed diagnosis, there were possibly or probably opportunities to improve diagnosis. Children with delayed diagnosis had worse outcomes. When the diagnosis was made, hospitalizations were longer, appendectomy was less likely, and appendiceal perforation and multiple abdominal surgical procedures were more likely. Taken together, our findings suggest that delayed diagnosis was more likely in patients with less classic presentations, that such patients were less likely to undergo imaging or receive appendicitis-directed treatments, and that a substantial fraction of delayed diagnoses were preventable despite being less clinically apparent.

Among children with delayed diagnosis of appendicitis, progression of disease occurred between the index and delay ED encounters. Nearly every classic symptom or sign of appendicitis was more likely to be present during the later visit. The PAS and pARC scores, which measure the likelihood of appendicitis, were higher during the delay encounter than during the index encounter. Abdominal guarding and migration of pain to the right lower quadrant were especially likely to develop after the index encounter: each feature was more than 10 times as likely to be present later. The presence of more classic symptoms during a second ED visit makes the diagnosis more discernible to clinicians. Conversely, the absence of classic appendicitis symptoms puts a patient at risk of a delayed diagnosis.

Nevertheless, in this study, most delayed diagnoses were probably or possibly MOIDs, indicating that there were potential opportunities to prevent delay. The 3 elements of the diagnostic process most associated with a MOID were failures in diagnostic reasoning that indicated that the patient’s history should have prompted a deeper evaluation. Most children with delayed diagnosis had neither a WBC count nor an imaging study performed at the index encounter. Children with a characteristic history or physical examination should undergo these tests in an effort to prevent delayed diagnosis. In this study, only 22.0% to 61.3% of children received indicated imaging based on a pARC score above the threshold at which imaging is cost-effective.^28^ This finding suggests an opportunity to improve detection.

Combined with previous work, our results suggest several ways to improve the detection of appendicitis. First, children with an intermediate or high probability of appendicitis should undergo peripheral WBC testing and ultrasonography to evaluate appendicitis.^23,28^ In this study, the WBC was obtained in only 24% of children with a delayed diagnosis of appendicitis. A visualized normal appendix on ultrasonographic examination in a child with a high clinical probability of appendicitis should typically prompt further diagnostic evaluation (such as cross-sectional imaging), as these ultrasonographic tests have a 19% false-negative rate.^17^ Second, although children with a low clinical probability of appendicitis should not undergo immediate imaging, longer periods of observation or peripheral WBC measurement could contribute to a correct diagnosis. Evolution of symptoms and additional diagnostic tests that increase case typicality may increase diagnostic accuracy.^29^ However, defining which clinical features should prompt further testing remains challenging, as patients with appendicitis may have only vague features early in their course.^18^ Nonetheless, our study suggests that evaluation of clinical decision support systems based on the pARC score may be a promising strategy to improve appendicitis diagnosis because many children with delayed diagnoses had pARC scores that should have prompted additional diagnostic work-up.^30^

### Limitations

Our study had several limitations. First, we included only pediatric hospitals, which differ from general hospitals in terms of resources (such as 24-hour ultrasonography and pediatric surgery availability), clinician background, and patient characteristics. Second, cases and controls were not drawn from the same population, which could lead to selection bias. However, this was unlikely to affect our results because the control institution’s case patient characteristics did not differ greatly from the other case institutions’ patients except in sociodemographic domains including race, ethnicity, and insurance. Unfortunately, differences between the source populations of cases and controls meant we could not evaluate the contribution of sociodemographic factors in delayed diagnosis, such as the racial disparities known to exist in appendicitis care.^11,31,32^ In addition, we could not evaluate whether patients had imaging performed at a transferring hospital before arrival to the index encounter. However, the overall very low rate of imaging in patients with delayed diagnosis compared with those with a timely diagnosis suggests that is unlikely to affect our findings.

## Conclusions

In this case-control study, children with delayed diagnosis of appendicitis initially had milder symptoms and signs, were likely to have progression of symptoms before diagnosis, and had worse outcomes than those with timely diagnosis. Our findings suggest that cases of delayed diagnosis of appendicitis in children may represent an early missed opportunity to make a diagnosis. Decision support interventions could be useful in preventing delayed diagnosis without leading to overtesting because many delayed diagnoses occur in cases where testing is indicated.

## Notes

Supplement. **eMethods.** Detailed Description of Imputation Methods **eTable 1.** Definitions of Delayed Diagnosis **eTable 2.** Comparison of Characteristics of Cases From the Control Site and the Other 4 Sites **eTable 3.** Association of Elements of Care With Preventability of Delayed Diagnosis of Appendicitis
