# Supplementary material for: Clinical Features and Preventability of Delayed Diagnosis of Pediatric Appendicitis
Source: JAMA Netw Open. 2021 Aug 31;4(8):e2122248. doi: 10.1001/jamanetworkopen.2021.22248 (PMC8408667; doi:10.1001/jamanetworkopen.2021.22248)
Supplement: Supplement. — eMethods. Detailed Description of Imputation Methods eTable 1. Definitions of Delayed Diagnosis eTable 2. Comparison of Characteristics of Cases From the Control Site and the Other 4 Sites eTable 3. Association of Elements of Care With Preventability of Delayed Diagnosis of Appendicitis [file jamanetwopen-e2122248-s001.pdf]

Supplementary Online Content

Michelson KA, Reeves SD, Grubenhoff JA, et al. Clinical features and preventability of delayed diagnosis of pediatric appendicitis. *JAMA Netw Open*. 2021;4(8):e2122248. doi:10.1001/jamanetworkopen.2021.22248

eMethods. Detailed Description of Imputation Methods
eTable 1. Definitions of Delayed Diagnosis
eTable 2. Comparison of Characteristics of Cases From the Control Site and the Other 4 Sites
eTable 3. Association of Elements of Care With Preventability of Delayed Diagnosis of Appendicitis

This supplementary material has been provided by the authors to give readers additional information about their work.

© 2021 Michelson KA et al. *JAMA Network Open.*

eMethods. Detailed Description of Imputation Methods

The white blood cell (WBC) count was not obtained in many case index encounters, presumably because appendicitis was not being considered as a diagnosis. A complete assessment of the clinical likelihood of appendicitis depends on a WBC count. Therefore, we imputed the WBC count using several methods of imputation including multiple imputation and using a range of WBC values including 5,000, 10,000, 15,000 and 20,000 cells/μL.

Multiple imputation was performed using linear regression using WBC as the outcome and the following covariates drawn from controls: sex, age, anorexia, pain with walking, guarding, migration of pain to the right lower quadrant, maximum pain in the right lower quadrant, duration of abdominal pain, and presence of perforated appendicitis. Using this model, we then generated a predicted WBC for patients with missing WBC. We did not impute absolute neutrophil counts; for the calculation of PAS, those without WBC did not get a point for elevated absolute neutrophil counts. For the calculation of pARC scores, we used the investigators' method for imputing ANC: $(-0.8783 + 1.1008 \times \sqrt{\text{WBC}}, \text{cells} \times 10^3/\mu\text{L})^2$

© 2021 Michelson KA et al. *JAMA Network Open.*

eTable 1. Definitions of Delayed Diagnosis

**Likelihood of delay**	**Definition**
Near-definitely not	(1) there were no signs of the condition on the prior encounter; (2) an alternative explanation for the prior encounter symptoms is definite or almost definite; or (3) the time course makes the condition virtually certain not to have been present.
Probably not	It is very unlikely that the condition was present on the prior encounter, and symptoms, signs, and other data at that time mostly pointed away from the condition.
Possibly	(1) it is possible the condition was present, but there are factors for and against that theory; (2) determination is confusing; (3) there is limited detail from which to decide; or (4) there are some alternative explanations for the case's features, but they have similar likelihood to the condition.
Probably	More likely than not, the patient had the condition on the prior encounter. Evidence pointed toward the condition, or few alternative explanations existed.
Near-definitely	The patient almost definitely or definitely had the condition on the prior encounter. Generally, these cases have clear evidence that the condition was present on the prior encounter. Alternatively, there is no other plausible explanation for the symptoms/signs on the prior encounter.

© 2021 Michelson KA et al. *JAMA Network Open.*

eTable 2. Comparison of Characteristics of Cases From the Control Site and the Other 4 Sites

	**Cases from control site N=50 (10.6%)**	**Cases from other 4 sites N=421 (89.4%)**	**p**
Age, years			0.87
<5	8 (16.0)	74 (17.6)	
5-9	19 (38.0)	166 (39.4)	
10-14	15 (30.0)	130 (30.9)	
15-21	8 (16.0)	51 (12.1)	
Male sex	28 (56.0)	203 (48.2)	0.37
Race			0.02
White	16 (32.0)	218 (51.8)	
Black	8 (16.0)	26 (6.2)	
Asian	0 (0.0)	3 (0.7)	
American Indian	0	0	
Pacific Islander	0 (0.0)	1 (0.2)	
Other	26 (52.0)	173 (41.1)	
Ethnicity			0.005
Hispanic or Latino	22 (44.0)	266 (63.2)	
Not Hispanic or Latino	25 (50.0)	150 (35.6)	
Unknown	3 (6.0)	5 (1.2)	
Insurance			<0.001
Public	23 (46.0)	273 (64.8)	
Private	23 (46.0)	133 (31.6)	
Self pay	0 (0.0)	15 (3.6)	
Unknown	4 (8.0)	0 (0.0)	
Complex chronic condition	5 (10.0)	36 (8.6)	
Abdominal pain duration			0.28
< 24 hours or unknown	0 (0.0)	16 (3.8)	
24 to 47 hours	19 (38.0)	199 (47.5)	
48 to 96 hours	17 (34.0)	96 (22.9)	
>96 hours	9 (18.0)	62 (14.8)	
Anorexia	5 (10.0)	46 (11.0)	
Fever	23 (56.1)	243 (78.4)	0.003
Nausea/vomiting	14 (28.6)	166 (41.4)	0.09
Pain with walking	41 (82.0)	316 (77.1)	0.48
Maximal pain in the right lower quadrant	8 (27.6)	57 (33.9)	0.67
Migration of pain to the right lower quadrant	10 (20.0)	95 (26.3)	0.39
Abdominal guarding	2 (4.3)	66 (29.2)	<0.001
Days between index and delay encounters, median (IQR)	1 (1-2)	1 (1-2)	0.92

© 2021 Michelson KA et al. *JAMA Network Open.*

Signs and symptoms were assessed at the index (first of two) encounter. Cases were drawn from five sites, but only one of them was used to identify controls. Differences in characteristics could introduce selection bias.

eTable 3. Association of Elements of Care With Preventability of Delayed Diagnosis of Appendicitis

Safer Dx item number	Safer Dx item description	Score, median (IQR)	Correlation with overall likelihood of MOID (ρ)
1	The documented history was suggestive of an alternate diagnosis, which was not considered in the diagnostic process.	2 (1-4)	0.215
2	The documented physical exam was suggestive of an alternate diagnosis, which was not considered in the diagnostic process.	1 (1-3)	0.226
3	Data gathering through history, physical exam, and review of prior documentation was incomplete, given the patient's medical history and clinical presentation.	1 (1-3)	0.245
4	Alarm symptoms or "red flags" were not acted upon.	3 (2-5)	0.646
5	The diagnostic process was affected by incomplete or incorrect clinical information given to the care team by the patient or their primary caregiver.	1 (1-1)	0.046
6	The clinical information should have prompted additional diagnostic evaluation through tests or consults.	4 (2-5)	0.768
7	The diagnostic reasoning was not appropriate, given the patient's medical history and clinical presentation.	3 (2-5)	0.768
8	Diagnostic data (laboratory, radiology, pathology, or other results) available or documented were misinterpreted in relation to the subsequent final diagnosis.	1 (1-4)	0.326
9	There was missed follow-up of available or documented diagnostic data (laboratory, radiology, pathology, or other results) in relation to the subsequent final diagnosis of appendicitis.	1 (1-1)	0.172
10	The differential diagnosis was not documented OR the documented differential diagnosis did not include the subsequent final diagnosis.	1 (1-4)	0.179
11	The final diagnosis (appendicitis) was not an evolution of the care team's initial presumed diagnosis (or working diagnosis).	5 (4-6)	0.252
12	The clinical presentation at the initial encounter was mostly typical of the diagnosis of appendicitis.	3 (2-5)	0.758
13	In conclusion, based on the above questions, the episode of care under review had a missed opportunity to make a correct and timely diagnosis.	4 (3-5)	1

© 2021 Michelson KA et al. *JAMA Network Open.*

Each item was rated by a trained reviewer on a scale of 1-7. Scores and correlations were only determined among cases (those with delayed diagnosis).

© 2021 Michelson KA et al. *JAMA Network Open.*
